# Comparative Study of Gelatin Hydrogels Modified by Various Cross-Linking Agents

**DOI:** 10.3390/ma14020396

**Published:** 2021-01-14

**Authors:** Joanna Skopinska-Wisniewska, Marta Tuszynska, Ewa Olewnik-Kruszkowska

**Affiliations:** 1 Chair of Biomaterials and Cosmetics Chemistry, Faculty of Chemistry, Nicolaus Copernicus University in Torun, Gagarina 7 Street, 87-100 Torun, Poland; marta.tuszynska96@gmail.com; 2 Chair of Physical Chemistry and Physicochemistry of Polymers, Faculty of Chemistry, Nicolaus Copernicus University in Torun, Gagarina 7 Street, 87-100 Torun, Poland; olewnik@umk.pl

**Keywords:** gelatin hydrogels, cross-linking, EDC-NHS, squaric acid, dialdehyde starch

## Abstract

Gelatin is a natural biopolymer derived from collagen. Due to its many advantages, such as swelling capacity, biodegradability, biocompatibility, and commercial availability, gelatin is widely used in the field of pharmacy, medicine, and the food industry. Gelatin solutions easily form hydrogels during cooling, however, the materials are mechanically poor. To improve their properties, they are often chemically crosslinked. The cross-linking agents are divided into two groups: Zero-length and non-zero-length cross-linkers. In this study, gelatin was cross-linked by three different cross-linking agents: EDC-NHS, as a typically used cross-linker, and also squaric acid (SQ) and dialdehyde starch (DAS), as representatives of a second group of cross-linkers. For all prepared gelatin hydrogels, mechanical strength tests, thermal analysis, infrared spectroscopy, swelling ability, and SEM images were performed. The results indicate that the dialdehyde starch is a better cross-linking agent for gelatin than EDC-NHS. Meanwhile, the use of squaric acid does not give beneficial changes to the properties of the hydrogel.

## 1. Introduction

Gelatin is a product of partial hydrolysis of collagen, the main structural protein of connective tissues. The gelatin molecular weight and properties depend on collagen source and method of its manufacturing-heat and enzymatic denaturation, or extraction in alkaline (gelatin type B) or acidic (gelatin type A) conditions [1,2]. The triple helical structure of collagen is irreversibly destroyed during this process, but molecular composition changes slightly. The repeating sequence of amino acids -Gly-X-Y- and the relatively high content of hydroxyproline and hydroxylysine typical for collagen remain as gelatin characteristic features [3,4]. However, depending on the method of gelatin preparation, various amino acids may be degraded. The most significant difference is the hydrolysis of asparagine and glutamine amide groups, resulting in lowering of the isoelectric point of gelatin type B [5,6,7].

Gelatin has a number of advantages that make it readily used in many areas such as food industries, in drug and cell delivery, for tissue engineering applications targeting several tissues such as bone, cartilage, and skin [8,9,10,11], and also in medicine as wound dressing, plasma expander, adhesives, and absorbent pads for surgical use [12,13]. Gelatin is water-soluble, easily commercially available, much cheaper than collagen, and at the same time, it still contains binding moieties important for cell attachment. The wide interest in gelatin is also caused by its non-toxicity, non-carcinogenicity, biocompatibility, and biodegradability. However, the gelatin-based materials are characterized by poor mechanical properties, thermal instability, and relatively short degradation time [7,14,15]. These disadvantages can be reduced by cross-linking the material.

Gelatin materials may be modified by physical and chemical methods, e.g., the use of plasma, UV radiation, and dehydrothermal treatment (DTH), combined freeze-drying/leaching method [16,17], the use of chemical cross-linking agents [14,18,19], or enzymes [5,8]. However, the chemical cross-linking method is still considered to be the most effective and the most popular approach to cross-link gelatin. The chemical cross-linking agents are usually divided into two groups. Non-zero-length cross-linkers react with amino or carboxyl groups of amino acids and are incorporated into a gelatin network structure. Several bi-functional or poly-functional cross-linking reagents have been used including aldehydes (such as glutaraldehyde, glyceraldehyde, and formaldehyde), polyepoxides, isocyanates, and natural products such as genipin [13,14,15,20]. Zero-length cross-linking is another example of a chemical cross-linking method. In this technique, carboxyl groups are activated by cross-linker to direct reaction with amino groups present on the adjacent gelatin chain. The reagent is not built into the gelatin matrix. One of the most well-used linkers of this type is 1-ethyl-3-(3-dimethylamino propyl) carbodiimide hydrochloride (EDC). It can be combined with *N*-hydroxysuccinimide (NHS), which increases the efficiency of the gelatin cross-linking reaction [8,13,15,16,19,20]. However, chemical cross-linking also has some limitations. For example, glutaraldehyde is a very effective, low-cost, and easy to use cross-linking agent, but its unreacted molecules remaining in the material may cause cytotoxicity. On the other hand, genipin is a much safer and equally effective cross-linker, but very expensive. Therefore, new, effective, safe, and economically acceptable cross-linking methods are constantly being sought [7,14,15]. For this reason, in our work, the gelatin was cross-linked with three different cross-linking agents: EDC-NHS, squaric acid (SQ), and dialdehyde starch (DAS).

1-ethyl-3-(3-dimethylamino propyl) carbodiimide hydrocholoride is the most popular carbodiimide used for coupling biological substances containing carboxyl groups and amines. This carbodiimide is known to be non-toxic and biocompatible [5,21]. EDC reacts with a carboxyl group and forms an intermediate that reacts with primary amino groups. The addition of NHS stabilizes the amine-reactive intermediate and significantly increases the efficiency of EDC-mediated cross-linking reactions [8,16,21,22]. The cross-linking agent rarely used is dialdehyde starch. DAS is obtained by selective periodate oxidative cleavage of the C2-C3 bond in starch monomer followed by two aldehyde groups formation. DAS is biodegradable and un-toxic, which makes it another class of valuable cross-linking agent [23,24]. Squaric acid, 3,4-dihydroxy 3-cyclobutene 1,2-dione, is a molecule with a cyclic, symmetrical, planar, and rigid structure. Squaric acid is a highly acidic molecule that exists in keto-enol balance [25]. Negative charges are evenly distributed in the molecule between the oxygen atoms in a completely symmetrical dianion. Therefore, squaric acid reacts readily with amino groups and may be incorporated into the polymer network. Squaric acid derivatives are used as substitutes for reagents commonly used in biology, which are among others monophosphates, other carboxylates, and diesters, used in many medical aspects [26,27]. Our previous researches showed that DAS and SQ are effective cross-linking agents for collagen hydrogels.

The aim of the study was to compare the properties of gelatin cross-linked by typically used cross-linker EDC-NHS with the material modified by the addition of other cross-linking agents: Squaric acid and dialdehyde starch.

## 2. Materials and Methods

### 2.1. Materials

Gelatin from porcine skin (type A, 300 Bloom), *N*-hydroxysuccinimide, 1-ethyl-3-(3-dimethylamino propyl) carbodiimide hydrochloride, and 3,4-dihydroksy-3-cyklobuten 1,2-dion were obtained from Sigma-Aldrich, Steinheim, Germany. Dialdehyde starch was purchased from CHEMOS GmbH, Altdorf, Germany. All the materials were used without any further purification.

### 2.2. Hydrogel Preparation

Forty percent aqueous solution of gelatin was prepared by mixing gelatin in the distilled water with a magnetic stirrer at 50 °C for 30 min. The cross-linkers solutions were prepared by dissolving a proper amount of EDC-NHS (in molar ratio 1:5) and SQ in distilled water in separate beakers. Dialdehyde starch was stirred in the water at 60 °C until the clear solution was obtained. After that, 15 cm^3^ of 40% aqueous solution of gelatin was mixed with the desired amount of different cross-linkers and refilled to 30 cm^3^ of total volume with distilled water to finally obtain a solution of 20% gelatin. One percent of DAS and EDC-NHS (relative to protein dry weight), and 1% and 3% of SQ were added to gelatin solution. Then, 25 cm^3^ of mixed solutions were poured on the bottom of levelled dishes (1 mm thickness of the liquid layer) and left to gelation.

### 2.3. Thermal Analysis

The thermal properties of unmodified and cross-linked gelatin materials were studied using the Simultaneous TGA-DTA NETZSCH Thermal Analysis TA Instruments type STA 449F5 Thermoanalyzer (NETZSCH, Selb, Germany) in the temperature range from 30 °C to 650 °C. Samples weighing about 10 mg were tested. The measurement was performed under a nitrogen atmosphere with a gas flow rate of about 50.0 mL/min. The heating speed was 10 K/min. The research program that was used to determine the loss of mass during material degradation and the temperature values at the maximum speed of the entire process was the NETZSCH Proteus software (Version 6.1.0).

### 2.4. Infrared Spectroscopy

The thin slides of gelatin gels were dried in air. The FTIR-ATR spectra of samples were recorded using a Thermo Fisher Scientific Nicolet iS10 FTIR spectrophotometer equipped with a Ge single crystal attachment (Thermo Fisher Scientific, Waltham, MA, USA). The spectra of all samples were recorded in the wavenumber range 4000–600 cm^−1^, with a resolution equal to 4 cm^−1^. Sixty-four scans were performed for each of the samples. The results were processed using the OMNIC program (Version 9.2.41).

### 2.5. SEM Images

The morphology images of lyophilized gelatin biomaterials were obtained with the use of a scanning electron microscope manufactured by LEO Electron Microscopy Ltd., Cambridge, UK, 1430 VP model. The morphology images of lyophilized gelatin biomaterials were obtained with the use of a scanning electron microscope manufactured by LEO Electron Microscopy Ltd., UK, 1430 VP model. The gelatin solutions with cross-linker additions were poured into a 12-well plate and left to gelation. Then the material was frozen at −20 °C and lyophilized. The small piece (3 mm × 3 mm) of the material was cut from the middle of the sample and coated with gold. The cross-section of the hydrogel was observed. The pore size was analyzed using a program provided by the SEM manufacturer. The value was an average of at least ten measurements.

### 2.6. Mechanical Properties

**Figure 1:**
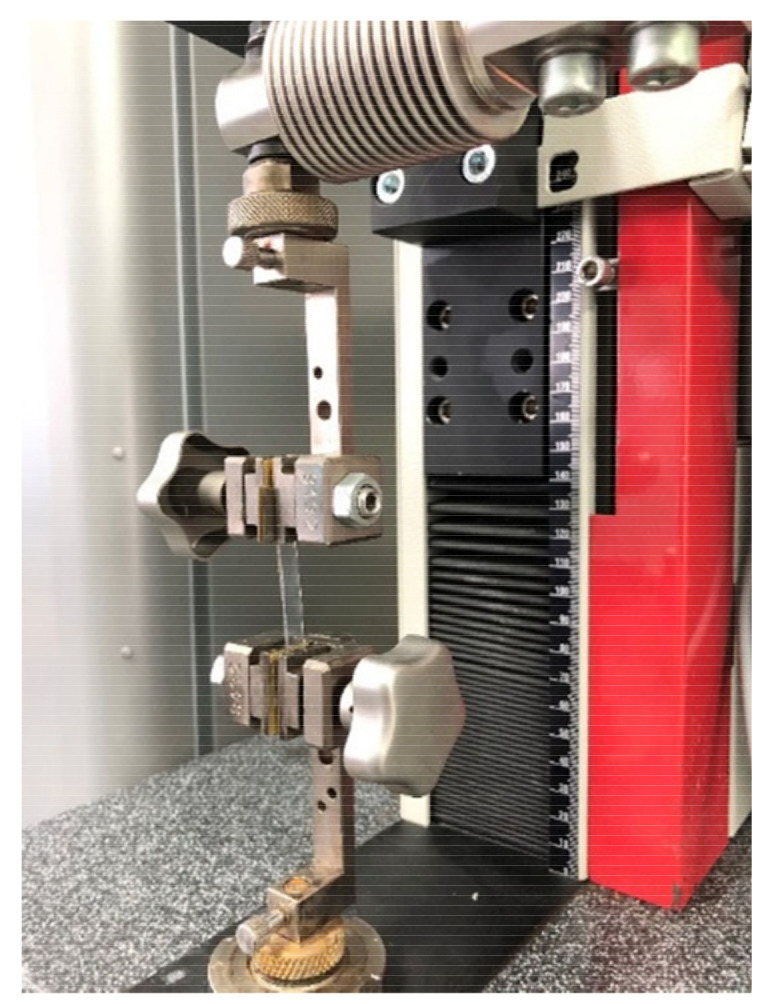
Photography of the Zwick & Roell Z 0.5 machine with measured hydrogel placed between the metal clamps.

The mechanical properties were determined using the Zwick & Roell Z 0.5 machine (Zwick&Roell, Ulm, Germany) (Figure 1). The material was cut with a die into equal strips, approx. 7.09 mm wide and 70 mm long. The thickness of each sample was measured using a caliper, approx. 1.18 mm. The ends of each strip were taped with sandpaper before measurement. The samples were placed between the metal clamps, carefully gripped, and kept lightly taut. The sample was stretched at a speed of 10 mm/min. Tensile strength, elongation at breaking point, and Young Modulus were determined. From each hydrogel, 7 strips were cut out, and the measurement was made for each until the sample was broken. The final results are the mean of at least 5 measurements, excluding two extreme results.

### 2.7. Swelling Ability

The swelling ability (E_s_) was measured by the conventional gravimetric method. The dry sample was weighed and placed in 3 mL of 0.05 M phosphate buffer saline (PBS) at pH 7.4, at room temperature. After the appropriate incubation time (1, 2, 4, 6, 24, 48, 72, 96 h), the excess of the phosphate buffer was removed with the use of absorbent paper, and the wet material was weighed (W_s_). The liquid content of the scaffolds was defined as the ratio of weight increase (W_s_ − W_d_) relative to the initial weight (W_d_) of dry samples. Each value was averaged from three parallel measurements. E_s_ was calculated with the following equation: $E_{s}=\left(W_{s}-W_{d}\right)/W_{d}$ where W_s_ and W_d_ denote the weights of swollen and dry samples, respectively.

## 3. Results

### 3.1. Thermal Analysis

**Figure 2:**
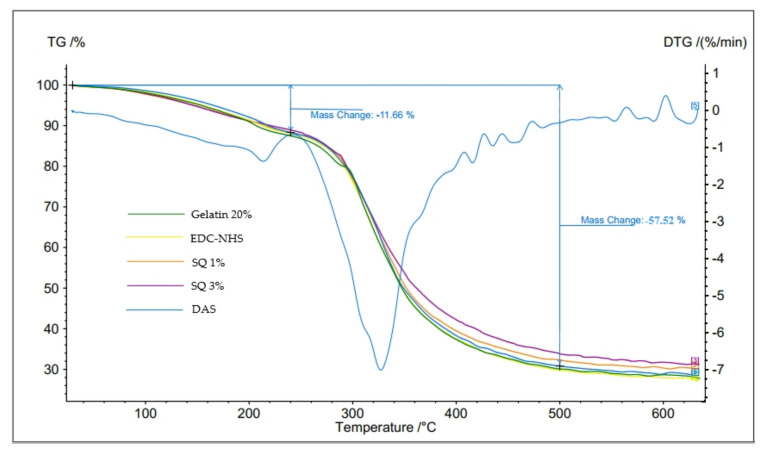
Thermograms of Gelatin 20%, as a model of mass change, and gelatin hydrogels cross-linked with EDC-NHS, squaric acid (SQ), and dialdehyde starch (DAS).

Generally, thermal stability is related to the cross-linking density of the polymeric material. Therefore, the thermal analysis of the obtained gelatin hydrogels, unmodified and cross-linked by various reagents, was carried out. All the hydrogels showed two steps of weight loss during heating (Figure 2). The first stage of gelatin hydrogels thermal decomposition occurred in the temperature range of 25–217 °C and was accompanied by weight loss from 11% to 12%. This stage was attributed to the loss of water absorbed by the hydrogel and water bound to gelatin. The second stage of hydrogel decomposition took place in the temperature range of 240–500 °C, with a weight loss from 66% to 70%. At this stage, the loss of innermost strongly bonded water molecules and protein chains decomposition are observed. The recorded temperature ranges of individual stages of thermal changes are consistent with the literature data [28,29,30].

The temperature of the changes and weight losses for individual samples were analyzed, and the results are presented in Table 1. The unmodified Gelatin 20% hydrogel showed the lowest temperature and the highest weight loss in the first stage of thermal degradation. The temperature of this step increased, and the amount of water loss decrease after the material cross-linking using EDC-NHS, SQ, and DAS. The structure of cross-linked hydrogel is more closed, therefore, it binds less water and the evaporation of water is more difficult. Additionally, the temperature of the second degradation stage was the lowest for Gelatin 20%, and the observed weight loss was 69%. It is well known that the thermal stability of protein materials is related to the total number of hydrogen bonds, cross-linking bonds, and other interactions between biopolymer chains [4]. Therefore, the increase in degradation temperature observed for EDC-NHS, SQ 1%, SQ 3%, and DAS indicates the cross-linking bond formation. Though somewhat surprising is the fact that the greater amount of squaric acid (3%) improved the thermal stability of the gel to a lesser extent than the 1% addition, and at the same time, the lowest weight loss was observed for this sample. Perhaps, due to the very polar nature of the SQ molecule and the ability to the formation of hydrogen bonds to water molecules, the mobility of protein chains is modified, and this may decrease the material thermal stability. Moreover, the electrostatic repulsion of protein functional groups may be modified. Among the tested materials, DAS cross-linked hydrogel showed the highest temperature of the second stage of thermal changes.

### 3.2. Infrared Spectroscopy

**Figure 3:**
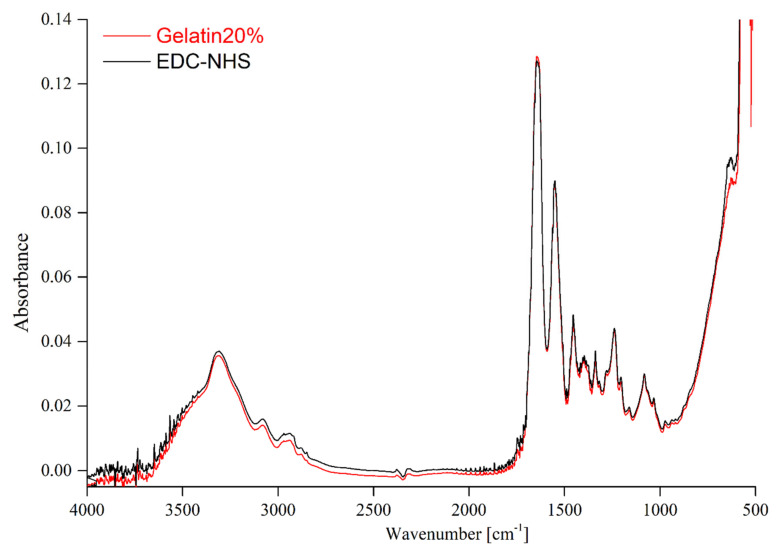
The FTIR spectra of gelatin and gelatin crosslinked by EDC-NHS.

**Figure 4:**
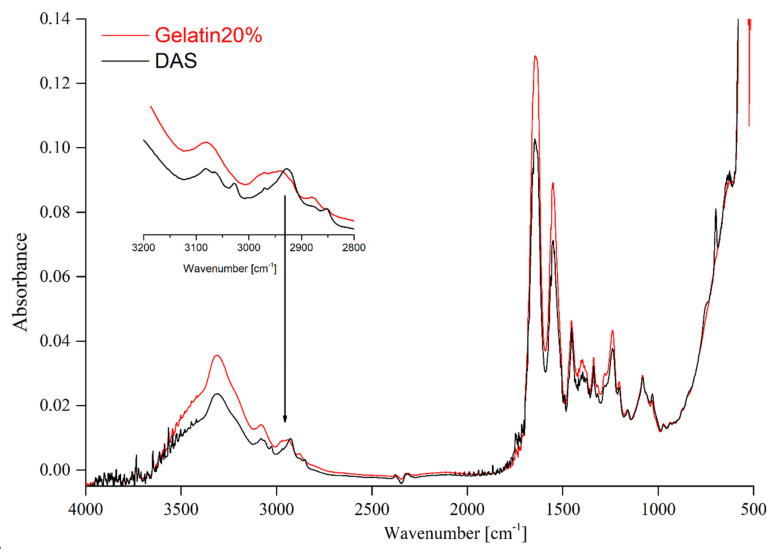
The FTIR spectra of gelatin and gelatin crosslinked by DAS.

**Figure 5:**
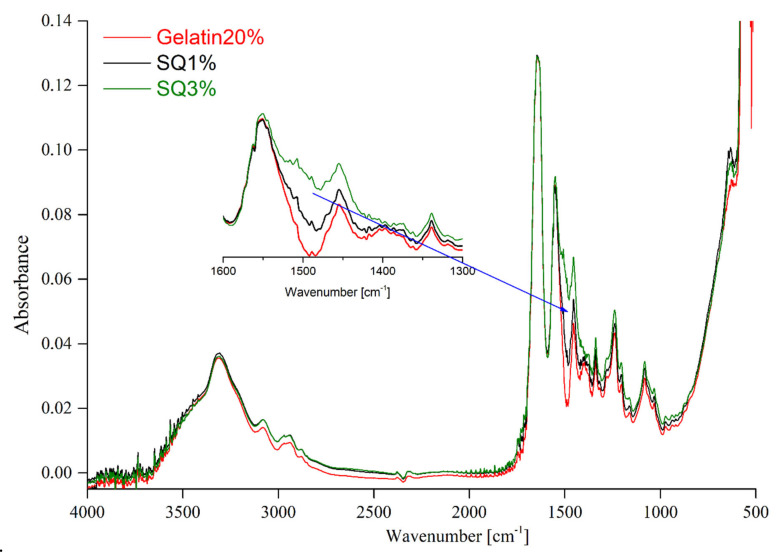
The FTIR spectra of gelatin and gelatin crosslinked by means of squaric acid.

In an attempt to establish the influence of the crosslinking agent on the structure of gelatin-based materials, the FTIR-ATR technique was applied. The FTIR spectra of pristine gelatin and gelatin-based films are presented in Figure 3, Figure 4 and Figure 5.

According to the published data, the obtained gelatin FTIR spectra indicate the presence of absorption bands characteristic of peptide bonds (Figure 3). In Table 2, the bands have been assigned to the characteristic groups of atoms. One of the most important groups in the structure of gelatin is the amide A with the band in the wavenumber range between 3500 and 3200 cm^−1^. Other characteristic groups present in the structure of gelatin include: The amide B band visible at the 3080 cm^−1^ wavenumber amide I, amide II, and amide III bands, that can be observed at the 1646 cm^−1^, 1550 cm^−1^, and 1239 cm^−1^ wavenumber, respectively [31]. The amide I band is attributed to the CO- and CN- stretching vibrations. The amide band II can be assigned to the deformation vibrations of the N-H group and the stretching vibration of C-N bonds. Additionally, other bands corresponded to the vibration stretching of the C-N bonds and the vibration of bending N-H bonds, indicating the presence of the amide III [4,10,15,25,32].

The spectra of unmodified gelatin and gelatin cross-linked using EDC-NHS are shown in Figure 3. EDC-NHS is a zero-length cross-linker, therefore the obtained results do not reveal changes in the structure of the obtained material. This suggests that the structure of the formed cross-linked gelatin-based film has not undergone any notable changes. The same tendency was observed in the publication by Dae Hoon Lee et al. [33]. A comparison of FTIR spectra of the pure gelatin and gelatin crosslinked by means of dialdehyde starch (DAS) (Figure 4) revealed certain differences in the regions that correspond to CH vibration stretching (2850–2950 cm^−1^) [23]. The presence of the band at 1032 cm^−1^ suggests that hydrogen bonds between gelatin chains and dialdehyde starch are created. When assessing the changes in the FTIR-ATR spectrum mentioned above, we have to bear in mind that the studied material contains as little 1% of DAS. Moreover, it has to be taken into account that gelatin and dialdehyde starch possess a large number of similar functional groups. Consequently, the otherwise simple observation of the crosslinking effect can be impeded by overlapping of the recorded bands [24,32]. In the case of materials consisting of gelatin and squaric acid (SQ), films with two different concentrations of the cross-linking agent were analyzed. The FITR-ATR spectra of pure hydrogel and gelatin containing 1 and 3% of squaric acid can be seen in Figure 5. The most significant changes between the crosslinked and non-crosslinked materials have been observed at the 1530–1480 cm^−1^ as well as the 650 cm^−1^ wavelength, which corresponds to the C=O stretching and C=O wagging vibrations present in the structure of the squaric acid [34].

### 3.3. SEM Images

**Figure 6:**
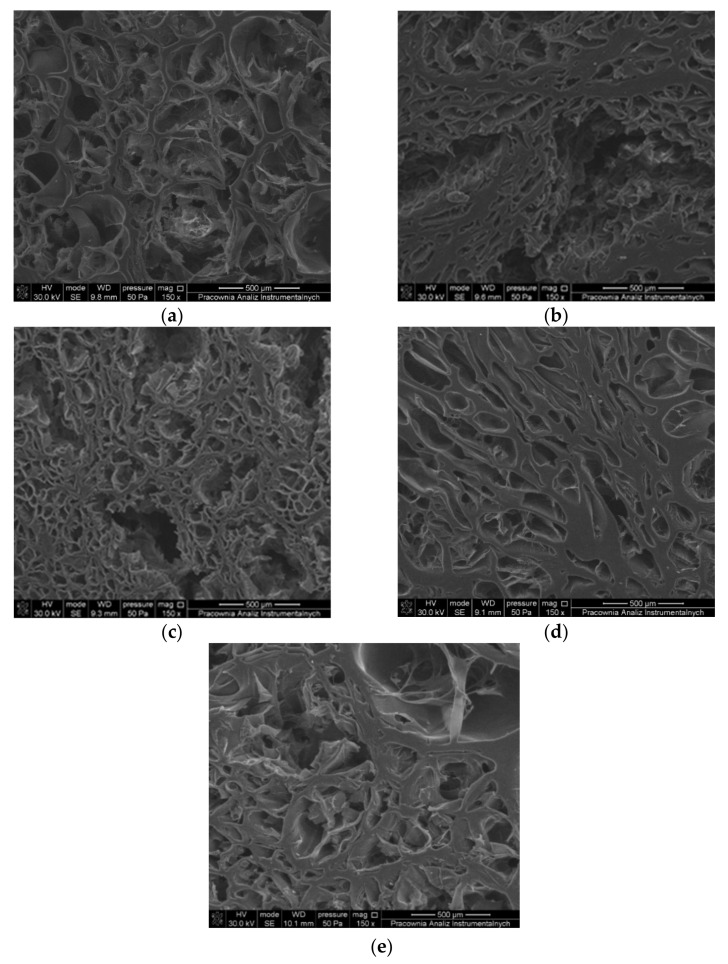
SEM images of lyophilized gelatin hydrogels: (**a**) Gelatin 20%, (**b**) EDC-NHS, (**c**) SQ 1%, (**d**) SQ3%, (**e**) DAS.

The porosity of hydrogel depends on the presence of cross-linking bonds. This parameter influences mechanical properties, swelling ability, and transport of active molecules across the gel structure. The SEM images of lyophilized gelatin hydrogels, unmodified and cross-linked with EDC-NHS, SQ, and DAS, are seen in Figure 6. All the materials possess a 3-D porous structure. The pores have an irregular shape and heterogenic size. However, it is seen that the pore size decreases after the use of cross-linking agents. The mean pore size in an unmodified gelatin gel is 244 μm (Table 3), while the smallest pores, about 73 μm in diameter, were observed in the gels EDC-NHS and SQ 1% hydrogels. Similarly to the thermal stability study, it was observed that the greater the addition of SQ resulted in less change of material properties. SQ 3% gel possess pores with an average size of 152 μm. We noted a similar effect when we investigated the cross-linking of collagen-elastin materials using different amounts of SQ. When a limit concentration of the cross-linking agent is exceeded, the size of pores present in the material increases [4,25,32]. The addition of DAS also causes a relatively small reduction in pore size, possibly due to the polymeric character of the cross-linker molecules.

### 3.4. Mechanical Properties

Hydrogels are a particular group of materials composed of two phases: A small amount of a porous polymer network constituting a solid phase, and a large amount of a liquid phase. These make the mechanical properties of hydrogels usually quite poor and limit the materials applications. These also make it difficult to test the mechanical properties of these materials and force a different approach to the analysis than in the case of classic polymer materials. The strength and flexibility of gels depend on the polymers’ composition and structure, cross-linking density, porosity, and water content. Despite many studies, researchers are still looking for better, more efficient, and biologically safe methods of cross-linking.

**Figure 7:**
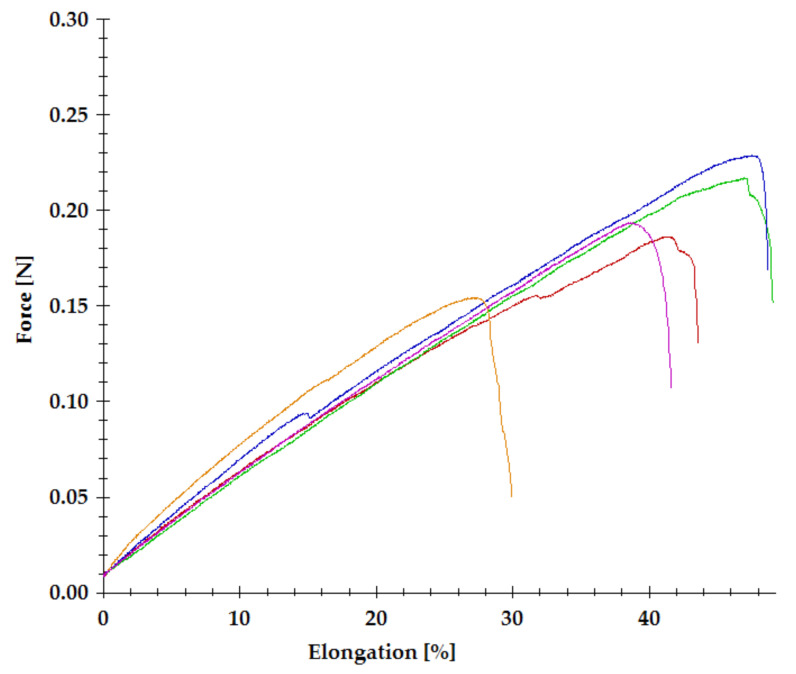
Stress-strain curve of gelatin hydrogel cross-linked with 1% SQ.

The obtained unmodified gelatin gels, and cross-linked by EDC-NHS, SQ, and DAS, were tested for tensile strength and elongation at breaking point, and then Young’s Modulus was calculated. The typical stress–strain curves of the cross-linked gelatin hydrogels are shown in Figure 7, and the calculated parameters are summarized in the diagrams presented in Table 4. Cross-linking with EDC significantly improves the mechanical properties of gelatin gels. The values of tensile strength and elongation at breaking point are twice as high as for unmodified gels. The stiffness of the material also increases (Young’s Modulus for EDC-NHS 99.43 kPa). A similar tendency is reported in the literature, though the results of mechanical tests cover a wide range of values. However, please note that the properties of gelatin materials depend on the gelatin type, its hardness, the procedure of the material formation (gel/film/fiber), and finally, the cross-linking degree [35,36,37,38,39]. Among the tested materials, the gelatin hydrogels cross-linked by DAS demonstrated the highest tensile strength. The value of elongation at breaking point comparable to Gelatin 20% and high Young’s Modulus proves the significant stiffness of the material. The results obtained for hydrogels with the addition of SQ were not as we expected. Our previous experiments showed a significant increase in the elastic modulus of collagen/elastin hydrogels cross-linked with SQ [25]. However, the tensile strength and elongation at breaking point decreased for both types of gelatin gels cross-linked by SQ. Only Young’s modulus slightly increased in case SQ 1%.

### 3.5. Swelling Ability

**Figure 8:**
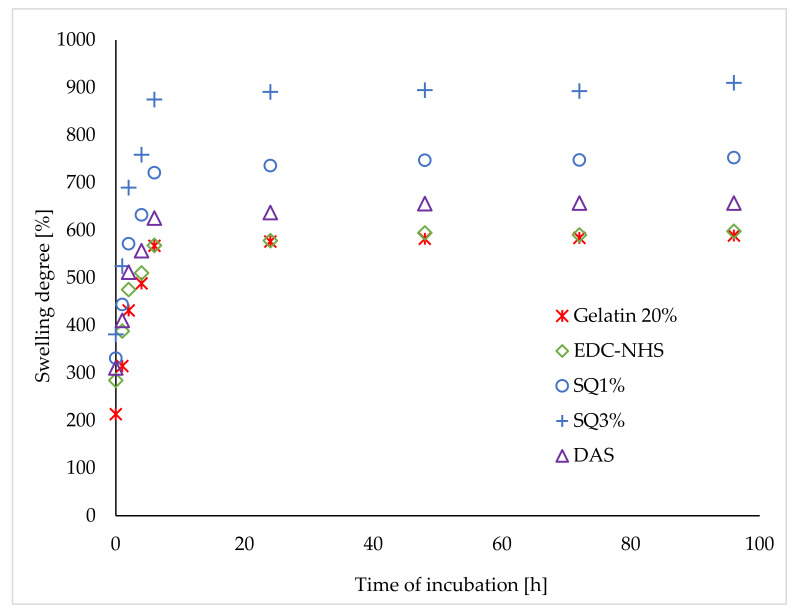
The swelling ratio E_s_ [%] of unmodified gelatin gels and cross-linked by EDC-NHS, SQ, and DAS.

A, high water absorption capacity of hydrogels is desirable due to the good permeability of such gels and their biocompatibility. The swelling curves of gelatin hydrogels cross-linked by various cross-linking agents are presented in Figure 8. Initially, the swelling degree of all the tested hydrogels increased rapidly, and then, after about 6 hours, it stabilized. Interestingly, the cross-linking did not cut the swelling capacity of hydrogels. A comparable swelling degree for Gelatin 20% and EDC-NHS gels was observed. The introduction of polar dialdehyde starch macromolecules and highly polar squaric acid into the hydrogel structure increased the swelling degree of the materials.

## 4. Discussion

The gelation of gelatin is a result of the conformational changes (coil-to-helix transition) and aggregation of the protein chains. The new structure, created during the cooling of the solution, is stabilized mainly by hydrogen bonds [3]. As a result, the gels have poor mechanical properties. Moreover, the hydrogen bonds can be easily broken on heating, making the gelatin gels thermally reversible. The material structure may be stabilized by introducing additional intermolecular covalent bonds by the cross-linking process.

EDC-NHS is a classical zero-length cross-linking agent. It mediates the formation of bonds between the amino and carboxyl groups, which makes the structure more rigid and close. Our experiments are consistent with previous results and prove that the cross-linking of the gelatin gel with EDC-NHS increases its thermal stability, rigidity, and mechanical strength of the material. As expected, the sizes of pores presented in the material were reduced threefold. The swelling capacity is comparable to the observed for Gelatin 20%, but this may be due to leaching unbound gelatin chains from the non-cross-linked gel structure [35,36,37,38,39].

**Figure 9:**
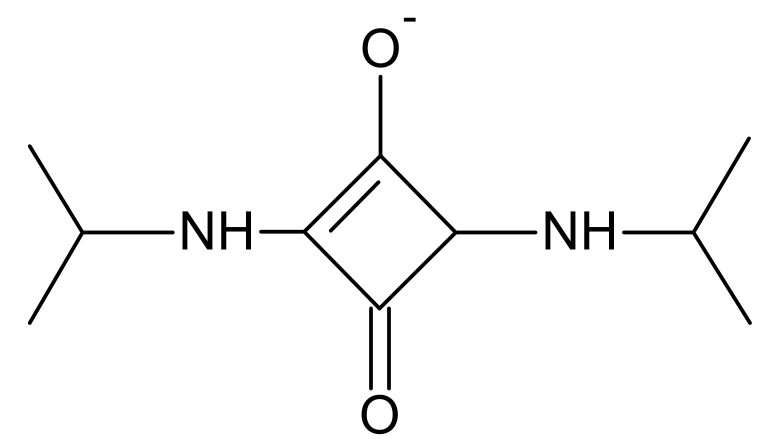
A scheme of the cross-linking bond between SQ and amino groups of a protein.

**Figure 10:**
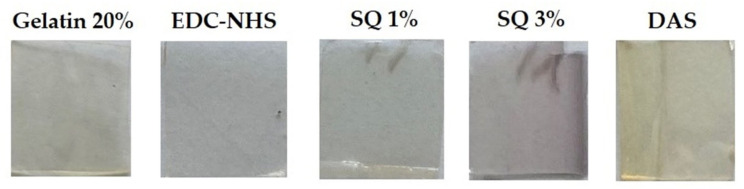
A comparison of color of the obtained gelatin hydrogels cross-linked by EDC-NHS, DAS, and SQ.

SQ is a representative of non-zero-length cross-linkers. It can form covalent bonds with amino groups of proteins and hydrogen bonds with carboxyl groups (Figure 9) [27,40]. The increase of thermal stability and additional bands observed in FTIR spectra indicates the formation of the cross-linking bonds. Moreover, the reduction of pore size in the material structure was also observed. However, in contrast to our earlier findings, which showed that higher SQ addition better improved the properties of collagen/elastin hydrogels, a larger SQ quantity changed the gelatin material properties to a lesser extent [25]. Interestingly, the addition of SQ increases the degree of hydrogel swelling. This phenomenon may be due to the very polar nature of SQ molecules, which can form hydrogen bonds with water molecules. Moreover, negatively charged squaric acid molecules can electrostatically repel charged areas of gelatin chains, and this promotes swelling. The most surprising was the significant deterioration in mechanical properties, especially with 3% SQ addition. It would be related to a very unusual phenomenon which we observed. Initially, all gels were light yellow, but after cutting strips for the mechanical test, the gels containing SQ 3% turned purple (Figure 10). Probably the cause of the color change was the contact with a metal punch. Cutting the hydrogel with other metal blades also caused similar color changes. Squaric acid is a substrate for the preparation of squaraine dyes [41,42]. Perhaps the contact with the metal initiated the reaction with electron-rich aromatic rings present in gelatin chains, and this led to the deterioration of the hydrogel mechanical properties.

**Figure 11:**
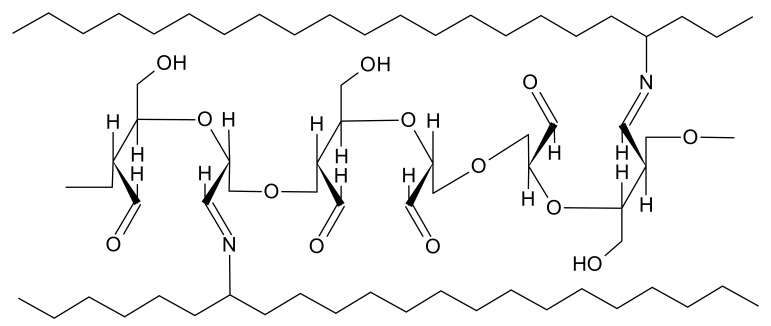
A scheme of the cross-linking bond between DAS and amino groups of a protein.

Dialdehyde starch is a macromolecular cross-linking agent with numerous aldehyde groups that can react with amino groups of gelatin (Figure 11). The cross-linking gelatin gel by DAS improves its thermal stability, as it was previously shown for collagen materials modified with DAS [32,43,44,45,46,47]. The tensile strength and stiffness of the materials significantly increase. Dou et al. have studied feather keratin films, and Martucci et al. gelatin films cross-linked by DAS [48,49]. They both observed a reduction of tensile strength value and an increase in the stiffness of the materials. Even though our results differ from these studies, they are consistent with those of Kaczmarek et al., who also observed strengthening and stiffening of lyophilized gelatin scaffolds cross-linked with DAS [24]. Although the structure of the gel cross-linked with dialdehyde starch is more rigid, we have observed an increase in the swelling capacity of materials containing DAS, similar to previous studies. It could be caused by the presence of numerous hydroxyl groups and the hydrophilic nature of DAS structure [24,32]. However, we have to bear in mind that various types of materials (films, porous scaffolds, gels) were tested, and various types and ratios of DAS were used as modifying agents. Therefore, it is difficult to directly compare the obtained results with the literature data [24,43,46,48,49].

## 5. Conclusions

In this paper, we examined the influence of three various cross-linking agents on gelatin hydrogel properties. The classical compound zero-length EDC-NHS, non-zero-length stearic acid, and macromolecular dialdehyde starch were tested. We have confirmed that EDC-NHS is an efficient cross-linking agent, which improves the mechanical properties and thermal stability of the protein gels. Contrary to expectations, SQ showed up as an ineffective cross-linking agent for gelatin-based gels. The reason for mechanical properties deterioration is probably the SQ transformation into squaraine dyes due to contact with a metal blade. Our results indicate that dialdehyde starch is the best cross-linking agent among tested compounds. It increases strength, stiffness, thermal stability, and interestingly, also the swelling capacity of gelatin hydrogels.

## Figures and Tables

**Table 1:** The parameters of the thermal decomposition of unmodified and cross-linked gelatin hydrogels.

Sample	I Stage 240 °C		II Stage From 240 °C to 500 °C	
	T [°C]	Δm [%]	T [°C]	Δm [%]
Gelatin 20%	204	12.47	305	57.38
EDC-NHS	212	11.87	316	58.34
SQ 1%	214	11.65	316	56.03
SQ 3%	217	11.04	309	55.12
DAS	214	11.66	326	57.52

**Table 2:** The position of main bands [cm^−1^] in FTIR spectra of unmodified and cross-linked gelatin hydrogels.

Sample	Amide A	Amide B	CH_3_	Amide I	Amide II	Amide III
Gelatin 20%	3308	3080	2940	1644	1551	1239
EDC-NHS	3307	3079	2940	1645	1550	1239
SQ 1%	3307	3080	2941	1646	1550	1239
SQ 3%	3307	3079	2943	1646	1550	1239
DAS	3307	3079	2970	1646	1550	1238

**Table 3:** The pore size of Gelatin 20% and gelatin hydrogels cross-linked with EDC-NHS, SQ, and DAS.

Sample	Pore Size (µm)
Gelatin 20%	243.63±35.98
EDC-NHS	73.35±5.64
SQ 1%	73.49±4.38
SQ 3%	151.72±26.70
DAS	130.08±16.44

**Table 4:** Mechanical parameters of the measured unmodified and cross-linked gelatin hydrogels.

Sample	Tensile Strength [kPa]	Elongation [%]	Young’s Modulus [kPa]
Gelatin 20%	31.68 ± 9.66	39.23 ± 2.98	37.60 ± 5.00
EDC-NHS	67.91 ± 7.13	74.77 ± 11.71	99.43 ± 11.83
SQ 1%	22.41 ± 6.30	40.54 ± 8.27	53.96 ± 4.34
SQ 3%	10.21 ± 1.60	37.71 ± 5.07	25.00 ± 3.39
DAS	111.91 ± 12.04	25.40 ± 2.84	168.00 ± 40.00

## Data Availability

The data presented in this study are available on request from the corresponding author.
